# The Immune Subtype Contributes to Distinct Overall Survival for Ovarian Cancer Patients With Platinum-Based Adjuvant Therapy

**DOI:** 10.3389/fimmu.2022.872991

**Published:** 2022-06-24

**Authors:** Yueyi Li, Hang Wang, Ming Chen, Xuelei Ma

**Affiliations:** ^1^ Department of Biotherapy, Cancer Center, West China Hospital, Sichuan University, Chengdu, China; ^2^ West China School of Medicine, West China Hospital, Sichuan University, Chengdu, China

**Keywords:** immune subtypes (ISs), ovarian cancer (OC), platinum, adjuvant therapy, overall survival (OS)

## Abstract

**Objective:**

Nowadays, platinum-based therapy has been widely used as the first-line therapy of ovarian cancer. However, the effect of the tumor microenvironment on platinum-based therapy remains unclear. In this study, we aim to investigate the relationship between immune microenvironment subtypes and the prognosis of platinum-based therapy in ovarian cancer.

**Methods:**

We integrated 565 ovarian cancer samples from two datasets and obtained the immune subtypes (ISs) by consistent clustering of 1190 immune-related gene expressions. The proportional hazards regression model was used to assess the relationship between ISs and the prognosis of platinum-based adjuvant therapy including progression-free survival (PFS) and overall survival (OS). The prognostic contribution of ISs was validated in three additional cohorts. Non-parametric tests were used to assess genomic characteristics, the proportion of immune cells, and immune-related signature differences among ISs.

**Results:**

We identified and validated five ISs associated with different clinical outcomes of the platinum-based adjuvant therapy in ovarian cancer patients. These differences were only found in OS rather than PFS. An immune subtype had the worst OS. Those patients mainly derived from the mesenchymal subtype had the lowest tumor purity with a high leukocyte fraction as well as stromal fraction and had the highest TGF-β response signaling. By contrast, an immune subtype characterized by immunoreactive status with the highest CD8+T cell infiltration and elevated IFN-γ response signaling had the best prognosis. Other subtypes with more diverse immunologic features such as lowest macrophage regulation signaling showed intermediate prognoses. Notably, the contribution of ISs to OS was independent of the clinical response to platinum-based drugs.

**Conclusion:**

Our analysis revealed the association between different immune characteristics and platinum-based adjuvant therapy, indicating the combination of ISs and chemotherapy could optimize the treatment strategy of OC patients.

## Introduction

Ovarian cancer (OC), is a complex gynecological disease that stands as the fifth main cause of cancer-related mortality among the female population with five-year survival rates below 45% (1). OC is capable of evading the immune system by pathological interaction between cancer cells and immune cells of the host within its tumor microenvironment (TME), which can therefore form an immunosuppressive network and promote tumor growth, so that further protect the tumor from the immune system (2). Nowadays, platinum-based therapy is used as the first-line therapy against OC (3). Due to the DNA-related damage caused by platinum drugs, tumors with abnormalities in DNA damage repair pathways would become more sensitive (4). Such a phenomenon is especially noteworthy for damage like homologous recombination repair deficiency (5, 6).

However, the mechanism of DNA damage failed to thoroughly illustrate the general prognosis picture of OC patients receiving platinum therapy. A portion of patients who have an initial response to the drug would gradually acquire drug resistance, which could therefore lead to a poor prognosis. In contrast, there is also a fair number of platinum-resistant patients who benefit from chemotherapy. Meanwhile, there were more advanced research outcomes coming up: some research teams found that the microenvironment was the main factor influencing the response to platinum-based chemotherapy (7). For the microenvironment of the tumor, effective T cells and fibroblasts stand as its main component. More specifically, CD8+ T cells from the effective T cells family help in reducing the friction by altering the metabolism of glutathione and cysteine in fibroblasts. Through a non-genetic mechanism, fibroblasts lessen the accumulation of platinum in OC cells, which further enhances the drug resistance against platinum-based therapy (8). Moreover, a two-way effect on the therapy response can contribute to the overall reaction of platinum-based treatment and help mediate cellular drug resistance (9, 10).

Previous studies managed to portray the immune landscape of the OC microenvironment (11, 12), yet the relationship between TME immunological features and the prognosis of patients receiving platinum-based adjuvant chemotherapy has been left undiscovered. In this study, we gather a large number of samples for identifying five robust ISs, therefore confirming that these subtypes with unique microenvironmental characteristics are indeed related to the prognosis of platinum-based therapy. Our results provide further evidence of TME in response to platinum-based chemotherapy and optimize the platinum-based treatment regimen for the better benefit of patients.

## Methods

### Data Sources

We obtained treatment information on OC samples from the TCGA database. And we acquired 253 OC patients who underwent platinum-based adjuvant chemotherapy after tissue collection based on the time of sampling and first treatment. Corresponding RNA-seq data with the format of fragments per kilobase million (FPKM) were obtained from UCSC Xena (http://xena.ucsc.edu). In addition, we collected 4 datasets of OC data sets that received platinum-based chemotherapy after surgery from GEO database: GSE32062 (n = 260), GSE63885 (n = 75), GSE30161 (n = 58), and GSE73614 (n = 107). Corresponding expression data and clinical follow-up information were downloaded from the Gene Expression Omnibus with the corresponding accession number (https://www.ncbi.nlm.nih.gov/geo). We combined TCGA-OV and GSE32062 datasets as discovery cohorts to identify ISs. And remaining cohorts were used to validate our results. The basic information about these patients was listed in **Supplementary Table S1**.

### Discovery of Immune Gene Modules and Subtypes

To ensure the reproducibility of the ISs, we integrated two independent gene expression datasets TCGA-OV and GSE32062. Due to the technical differences between the two data sets (RNA-seq and Microarray), we first performed batch correction of expression profiles using the ComBat method. Then we curated a comprehensive set of the immune-related gene from ImmPort (https://www.immport.org/shared/), of which 1190 genes had expression data in both two cohorts. After curating the immune-related gene profiles, we used a consensus clustering algorithm to identify immune gene modules (GMs) and ISs. Such process applied hierarchical clustering with the Euclidean distance metric and performed 500 bootstraps each encompassing 80% samples. The number of clusters varied from 2 to 10, and the optimal partition was determined by evaluating the consensus matrix and the consensus cumulative distribution function. The GO biological functions of each gene module were annotated and the top 10 terms of each GM were clustered based on semantic similarity. This analysis used the “clusterProfiler” and “ggtree” packages (13). Activity for gene modules was defined as the average expression level of genes in the corresponding module.

### Assign ISs in Additional Cohorts

We used two approaches to extend our subtyping system to additional data sets. The first method was based on clustering similarity. In three independent cohorts, we first calculated the activity of four gene modules, then used hierarchical clustering to identify subtypes. For each immune subtype in the discovery and validation cohorts, we generated the centroids of these gene module activities, and finally assigned ISs based on the correlation between centroids of validation and discovery cohorts. Since IS3 lacked typical gene module activity characteristics, the subtype assignment was mainly in IS1, IS2, IS4, and IS5. However, this method may lead to the fuzzy assignment of the subtypes in the validation datasets.

Therefore, we designed a multinomial logistic regression model in the TCGA-OV cohort to identify the subtypes in validation cohorts. Moreover, we evaluated the effectiveness of the model based on the area under ROC and PRC (precision-recall curve) in the GSE32062 dataset. To avoid data leakage, we tested the clinical applicability of this model with additional datasets (GSE63885, GSE30161, and GSE73614). These analyses were carried out using the “nnet” and “modEvA” R packages. This model can also reflect the importance of different gene modules for each subtype, which may reduce the influence of feature mixing on immune subtype recognition.

### Immune-Related and Genomic Features of Ovary Cancer Sample

We calculated expression signatures including proliferation, macrophages/monocytes, overall lymphocyte infiltration, TGF-β response, IFN-γ response, and wound healing. The composition ratio of 22 immune cells in the sample was inferred using the CIBERSORT algorithm (14). These proportions were multiplied by leukocyte fraction to yield corresponding absolute proportion estimates. The leukocyte fraction was estimated from a mixture model with specific methylation probes. The ABSOLUTE algorithm was used to calculate tumor purity, aneuploidy scores, and ITH. The stromal fraction was defined as the total non-tumor cellular component, obtained by subtracting tumor purity from unity. TCR and BCR diversity measurements and relative abundance were collected by Thorsson et al (15).

### Statistical Analyses

We used Kruskal–Wallis test, Wilcoxon rank-sum test, Fisher’s exact test, or Chi-square test, as means of assessing associations between variables. Multiple pairwise comparisons following a significant Kruskal-Wallis test were performed by Dunn’s test. The p-values were adjusted for multiple testing using the Bonferroni method. The Kaplan–Meier method was performed for the visualization and the differences between survival curves were calculated by a log-rank test. Multivariate analysis adjusting for clinical parameters was determined through a Cox proportional hazards model. All statistical analyses were performed with R software (http://www.R-project.org).

## Results

### Revisiting ISs in OC Based on Functional Gene Modules

**Figure 1:**
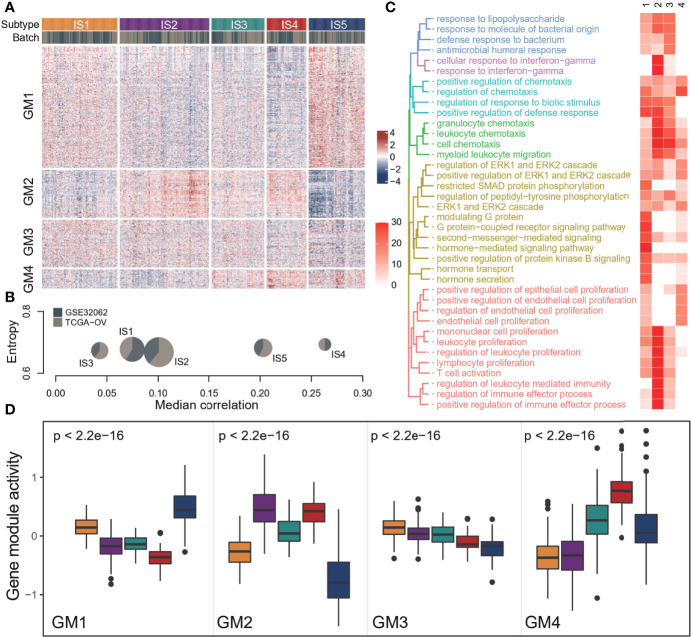
The landscape of immune subtype of ovarian cancer. **(A)** Cluster heat map of expression levels of 1190 immune‐related genes in 565 ovarian cancer samples. Column and row clusters represent ISs and gene modules. The data source for the sample was also annotated at the top of the heatmap. **(B)** Cohort distribution and average correlation between samples of each immune subtype. **(C)** The top 10 biological processes involved in each gene module, and colors in the heatmap indicate the significance of pathway enrichment (-log10(p)). **(D)** Expression patterns of four gene modules across five ISs. The middle bar in each box represents the median expression level of corresponding gene module activity in a certain immune subtype. In the boxplot, the yellow represents IS1, the purple represents IS2, the green represents IS3, the red represents IS4, and the blue represents IS5.

To better understand the immune landscape of OC, we integrated two independent data sets to systematically identify immune types (**Supplementary Figures S1A, B**). We obtained 4 immune GMs and 5 ISs from 565 samples based on consensus clustering (**Figure 1A**, **Supplementary Figures S1C, D, Supplementary Table S4**). The distribution of each immune subtype in the two data sets was relatively uniform, suggesting a stable result (**Figure 1B**). Different immune-related gene modules were associated with specific biology processes (**Figure 1C**, **Supplementary Table S7**). Specifically, we identified a gene module (GM1) strongly associated with hormone signaling pathways. GM2 had a strong immune effect, which was related to lymphocyte proliferation and activation, chemokine regulation, and IFNg response. GM3 was associated with antimicrobial response and GM4 with the regulation of epithelial cells and possibly weak immune activity.

Each immune subtype had a distinct expression pattern of immune gene modules (**Figure 1D**). In general, there was a significant difference in all GMs activity among subtypes, but the different degrees of GM3 was not as obvious as in the other three modules. IS1 had high GM3 activity and low GM4 activity. IS2 had high GM2 activity and low GM4 activity, suggesting a better-immunoactivated phenotype. By contrast, IS4 showed high GM4 activity and low GM1 activity. And IS5 showed high GM1 activity and low GM2 and GM3 activity. However, the GMs activity of IS3 was moderate, suggesting the lack of distinguishing features.

### Patients With Different ISs Have Diverse OS After Receiving Platinum-Based Adjuvant Therapy

**Figure 2:**
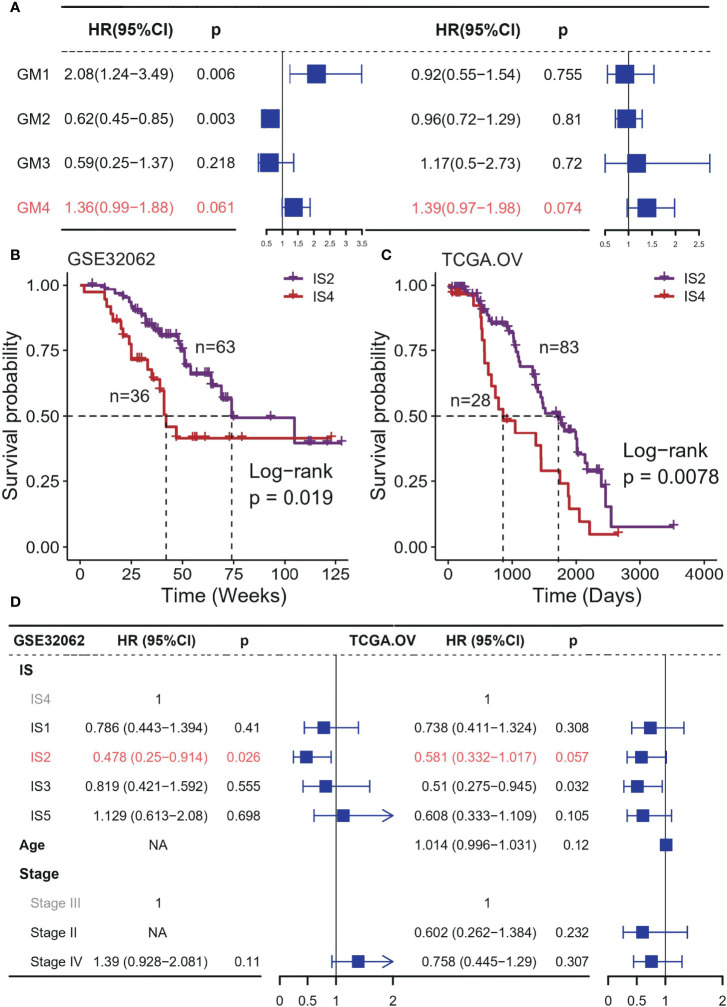
Association between ISs and prognosis in platinum-based adjuvant therapy in discovery cohorts. **(A)** Forest plot of the prognostic effect of gene module scores on predicting OS in GSE32062 (left) and TCGA (right) cohort. Kaplan–Meier curves show differences in OS for IS2 and IS4 patients in the **(B)** GSE32062 and **(C)** TCGA cohorts. **(D)** Multivariable Cox regression analysis of OS including immune subtype, stage, age in GSE32062 (left) and TCGA (right) cohort. IS 4 was used as the baseline for survival risk comparison for the immune subtype variable. Stage III was used as the baseline for survival risk comparison for the stage variable.

Then, we assessed the contribution of ISs to the prognosis of platinum-based adjuvant therapy. All patients in this analysis received adjuvant therapy with platinum-based drugs after surgery (**Supplementary Table S1**). Among four immune-related gene modules, GM4 activity was a risk factor for OS with a marginal significance in both two datasets (GSE32062: HR = 1.36, 95% CI 0.99-1.88, P = 0.061; TCGA: HR = 1.39, 95% CI 0.97-1.98, P = 0.074; **Figures 2A, B**). GM1 and GM2 were associated with poor and improved OS respectively in the GSE32062 cohort, but this pattern was not found in the TCGA dataset. These results were not stable in additional datasets (**Supplementary Table S5**), and the activities of all immune-related gene modules was not associated with PFS (**Supplementary Figure S2**).

We also observed significantly prognostic impact of the ISs in both TCGA and GSE32062 cohort, but only for OS (GSE32062: p = 0.033, log-rank test; TCGA: p = 0.035, log-rank test; **Supplementary Figures S3A, B**), and PFS excluded (GSE32062: p = 0.42, log-rank test; TCGA: p = 0.44, log-rank test; **Supplementary Figures S3C, D**). In general, there were significant differences in OS between IS2 and IS4, representing the best and the worst survival respectively (GSE32062: p = 0.019; TCGA: p = 0.44; log-rank test; **Figures 2B, C**). This survival difference was independent of some clinical factors (GSE32062: HR = 0.478, 95% CI 0.25−0.914, P = 0.026; TCGA: HR = 0.478, 95% CI 0.332−1.017, P = 0.026; **Figure 2D**). The remaining subtypes had intermediate prognoses, with no significant difference between them.

### The Associations of ISs and Prognosis in Platinum-Based Adjuvant Therapy Are Validated in Additional Cohorts

**Figure 3:**
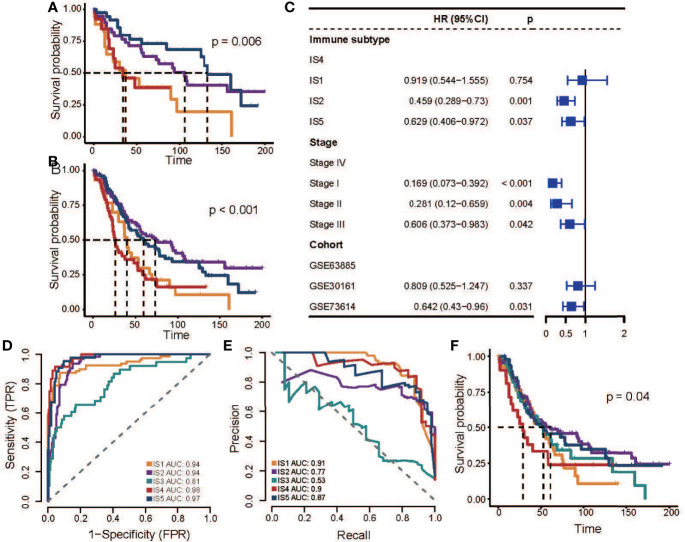
Association between ISs and prognosis in platinum-based adjuvant therapy in validation cohorts. **(A)** Kaplan–Meier curves for OS of GSE73614 stratified by the ISs. **(B)** Kaplan–Meier curves for OS of all patients in three independent cohorts stratified by the ISs. **(C)** Multivariable Cox regression analysis of OS including immune subtype, stage, and datasets in a combined validation cohort. IS4 was used as the baseline for survival risk comparison for the immune subtype variable. Stage IV was used as the baseline for survival risk comparison for the stage variable. GSE63885 was used as the baseline for survival risk comparison for cohort variables. **(D)** Receiver operating characteristics (ROC) curve of the multi-classification logistic model. **(E)** Precision-recall curve (ROC) of the multi-classification logistic model. **(F)** Kaplan–Meier curves for OS of all patients in three independent cohorts stratified by the multi-classification logistic model. In the figure, the yellow line represents IS1, the purple line represents IS2, the green line represents IS3, the red line represents IS4, and the blue line represents IS5.

Next, we validated this finding in three additional OC datasets that received platinum-based adjuvant chemotherapy after surgery. By calculating the average gene expression of four GMs, we performed hierarchical clustering of samples and assigned ISs based on GMs activity patterns and the correlation between discovery cohorts (**Supplementary Figure S4; Supplementary Table S3**). In the GSE30161 and GSE73614 cohort, we found an association between ISs and OS (GSE30161: p = 0.031; GSE73614: p = 0.006; log-rank test; **Figure 3A**, **Supplementary Figures S5A, B**), especially the difference in prognosis between IS2 and IS4. In the GSE63885 dataset, the pattern of difference in prognosis between IS4 and IS2 also existed, though it was not statistically significant (p = 0.215, log-rank test; **Supplementary Figure S5C**). We speculated that it might be due to the small sample size, which led us to further combine the samples of these three datasets and analysis again. The results showed that there was a significant difference in OS between IS2 and IS4 (p < 0.001, log-rank test; **Figure 3B**), and this result was not correlated with other factors such as the data set and stage (HR = 0.459, 95% CI 0.289−0.73, p = 0.001; **Figure 3C**, **Supplementary Figure S6**), which could further confirm the strong robustness of this pattern.

Then we developed a multi-classification logistic model based on TCGA data (Table S6) and verified the effectiveness of the model in the GSE32062 data set (**Figures 3D, E**). The area under the ROC indicates that the model has a good performance in identifying various subtypes (**Figure 3D**). Due to the imbalance in the sample proportion of each subtype, we further evaluated the accuracy and recall rate of the model (**Figure 3E**), and we found that the model had good precision in prediction except for IS3, which also indicated the ambiguity of IS3 subtype allocation. Finally, we evaluated the clinical potential of this model in additional pooled datasets (**Figure 3F**, **Supplementary Figure S7, S8**), and the results showed that the subtypes predicted by this model were also associated with patient outcomes, and the analysis result demonstrates that IS4 subtypes were associated with poor outcomes.

### The Effect of ISs on Prognosis Is Independent of the Clinical Response to Platinum Drugs

**Figure 4:**
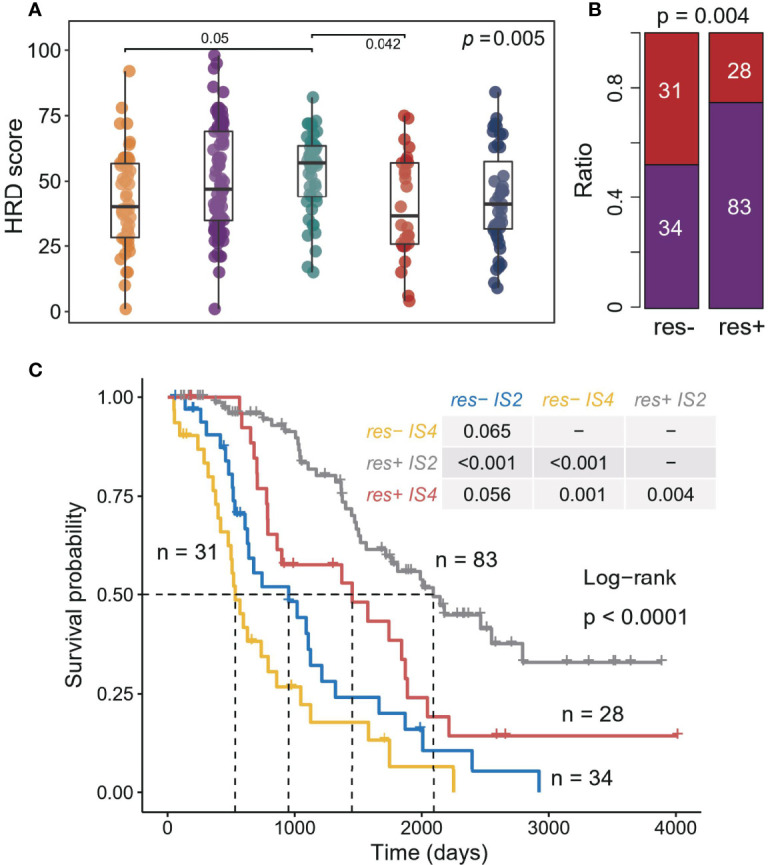
Association between ISs and response in platinum-based adjuvant therapy. **(A)** Homologous recombination deficiency score associated with the ISs. The significance of the Kruskal-Wallis test is marked on the top right. Multiple pairwise comparisons are performed by Dunn’s test, and only significant results are marked in the diagram. **(B)** Correlation between treatment response (complete response and incomplete response) and ISs (IS2 and IS4). The significance of the Chi-square test is marked at the top of the graph. **(C)** Kaplan–Meier curves for OS of all patients in three cohorts stratified by combining treatment response and ISs.

Furthermore, we explored the relationship between ISs and clinical treatment response. A total of three data sets recorded the clinical response of patients after receiving platinum-based chemotherapy (**Supplementary Table S1**), and the patients with complete response (CR) to platinum-based therapy strongly contributed to a better prognosis (**Supplementary Figure S9**). Previous studies showed that HRD scores can predict platinum drug response to a certain extent (16). Overall, the IS4 sample had the lowest HRD scores, but the difference in HRD scores between IS2 and IS4 (**Figure 4A**) was not significant. Moreover, IS2 patients tended to have more complete responders (p = 0.004, chi-sq test; **Figure 4B**). When considering ISs of patients, IS2 patients also had longer OS than IS4 patients, regardless of whether they responded to platinum (**Figure 4C**, **Supplementary Figure S10**), reflecting the promoting effect of the immune environment on treatment. These results suggest that immunological features have additional prognostic information, which could provide more guidance for platinum-based therapy and benefit more patients.

### ISs Associated With Prognosis Manifest Unique Molecular and Cellular Characteristics

**Figure 5:**
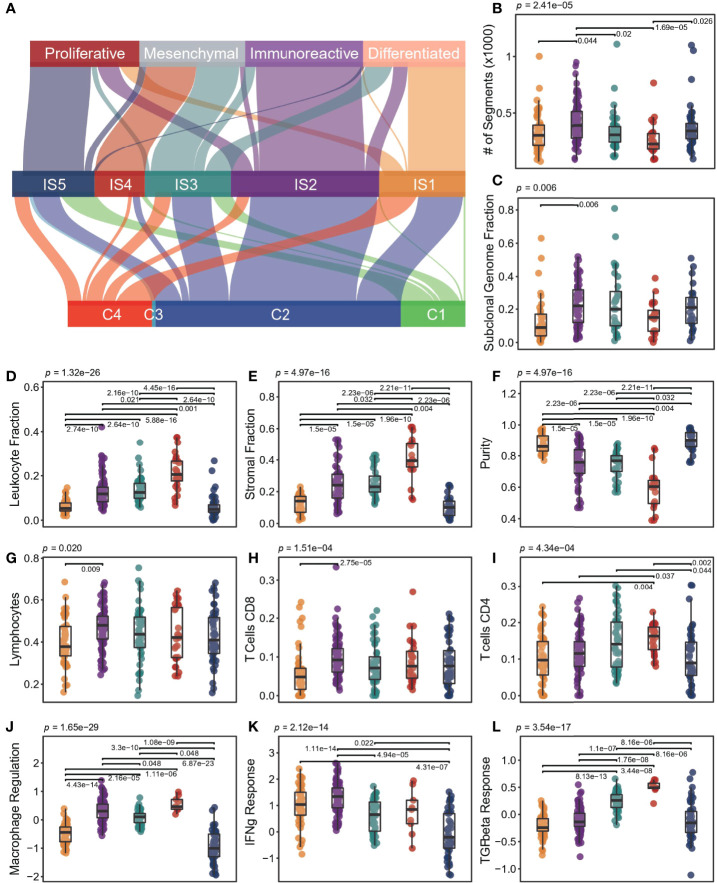
Molecular and cellular characteristics for ISs. **(A)** The distribution of patients was stratified by our ISs and the other subtypes proposed by previous research. **(B-L)** Different molecular and cellular characteristics associated with the ISs. The significance of the Kruskal-Wallis test is marked on the top. Multiple pairwise comparisons are performed by Dunn’s test, and only significant results are marked in the diagram. In the boxplot, the yellow represents IS1, the purple represents IS2, the green represents IS3, the red represents IS4, and the blue represents IS5.

A previous study discovered six immune classes across pan-cancer atlas based on immune-related signatures (15), where vast OC was divided into three subtypes (C1: wound healing, C2: IFN-g dominant, and C4: lymphocyte depleted). Our results recombined the previous ISs but had a high degree of consistency with transcriptional subtypes (17) (**Figure 5A**). IS1 was mainly derived from the differentiated type, where IS5 was from the proliferative type, and IS2 was from the immunoreactive type and IFN-g dominant type, whereas IS4 and IS3 were both from the mesenchymal type. By analyzing the association between these two typical OC subtyping systems and OS, we found that our subtyping system provided more prognostic information (**Supplementary Figure S11**).

At the genomic level, ISs were associated with intratumoral heterogeneity, copy number variation, and aneuploidy (**Figures 5B, C**). IS2 had more copy number variation, especially when compared with IS4 (p < 0.001, Wilcox test; **Figure 5B**). There was no significant difference in silent and non-silent mutation rates among subtypes, but IS4 tended to have a higher SNV neoantigen load (**Supplementary Figure S12**).

We observed significant differences in leukocyte fraction, stromal fraction, and tumor purity among ISs (**Figures 5D-F**). IS4 had the lowest tumor purity, with the highest leukocyte and stroma fraction, whereas IS1 and IS5 held the opposite. IS2 showed higher levels of M1 macrophages and lymphocytes, especially in CD8 T cells and Treg cells (**Figures 5G, H**, **Supplementary Figure S9**). However, IS4 had higher memory and activated CD4 cells, and lower helper T cells (**Supplementary Figure S9**). In addition, IS1 had higher T cells but lower CD4 T, CD8 T, and Treg cells (**Supplementary Figure S13**). Interestingly, IS5 showed more naive cells, including M0 macrophages and naive CD4 T cells (**Figure 5I**, **Supplementary Figure S13**). In terms of immune-related signatures, both IS2 and IS4 had high lymphocyte infiltration signals, but IS4 has the highest TGF-beta response signal, and IS2 has the highest IFN-g response signal (**Figures 5K, L**). In contrast, IS1 and IS5 had lower lymphocyte infiltration and macrophage regulation signals (**Figure 5J**), while IS5 had lower IFN-g signals (**Figure 5K**). Overall, IS3 had intermediate levels of immune features. In addition, we compared the expression levels of immune checkpoint molecules in different ISs (**Supplementary Figure S14**). The expression of PD1, PDL1, CTLA4, and LAG3 in IS2 was significantly higher than that in other subtypes, indicating that patients in IS2 may benefit from immune checkpoint inhibitor therapy.

## Discussion

Platinum-based adjuvant therapy is the main treatment for OC, and its therapeutic effect varies greatly among different patients. Hence, we systematically assess the influence of immune characteristics on the prognosis after platinum-based adjuvant therapy, attempting to find the protective effect of an immunoreactive subtype on treatment.

We identified four functional gene modules in integrated datasets. GM4 activity contributed to a poor prognosis and was associated with epithelial proliferation regulation and weaker immune function. It indicated that GM4 was involved in the process of epithelial-interstitial transition, promoting the progression and drug resistance of OC (18, 19). The activity of GM2 was associated with stronger immune function, such as lymphocyte proliferation and activation, chemokine regulation, and IFNg response. The activity of this module had a protective effect on prognosis in one dataset, suggesting that immune activation may contribute to platinum-assisted therapy. Conversely, another module in one dataset was a risk factor and was associated with the regulation of signaling pathways that have been suggested to be related to the occurrence and progression of OC (20). GM3 showed no significant association with prognosis. In general, the association between these gene modules and prognosis was in poor consistency or statistical margin in different cohorts, So we classified patients based on the activity of these modules and explored the association between immune microenvironment characteristics and platinum therapy from an overall perspective.

We identified five ISs that were associated with the prognosis of platinum-assisted therapy in discovery cohorts. Except for IS3, with no typical characteristics of gene module activity, the contribution of subtypes to prognosis was validated in three additional cohorts. IS3 had no unique characteristics of the immune microenvironment, providing limited prognostic information. This group of patients may be a mixture of other subtypes, which cannot be found independently in the small datasets, thus can be divided into some dominant subtypes. Resulting from differentiated and proliferative OC, IS1 and IS5 had a moderate prognosis, respectively (17). However, there was no significant difference in the immune microenvironment status between the two subtypes, which showed higher tumor purity and weaker immune infiltration level.

In contrast, IS2 and IS4 showed a strong correlation between the characteristics of the immune microenvironment and prognosis. Previous studies have shown that cancer-associated fibroblasts (CAFs) in the tumor stroma, a signal of tumor progression, protected ovarian tumor cells from platinum-induced apoptosis (8). Tumor growth in mice co-injected with CAFs and tumor cells was not inhibited by platinum. At the same time, IFN -γ signal eliminated the protective effect of CAFs on tumor cells and eliminated stroma-induced Pt resistance (8). In addition, TGF- Beta has been shown to induce osteopontin expression and secretion in mesenchymal cells. Its further activating downstream signals lead to chemotherapy resistance in OC (21). The association of these immune microenvironment interacts with platinum sensitivity was confirmed in these two subtypes of OC. Our results found that IS4 patients, with the highest stromal cell ratio and the strongest TGF-beta signaling, had the worst prognosis after platinum-based adjuvant therapy, and IS2 patients with the strongest IFN-γ signal showed the best prognosis.

Interestingly, we found that the association between ISs and platinum therapy was solely limted to OS, but not for PFS, suggesting that the effect of the immune microenvironment on efficacy is durable. This association was independent of the patient’s response status to the drug. Patients in the IS4 group were able to achieve relatively good outcomes even with poor treatment responses.

There is one limitation of this study that should be noted here. Some samples in our cohorts have experienced the combined drug treatment (platinum combined with cyclophosphamide, taxane, etc). As a large number of samples without detailed treatment course, we were unable to evaluate whether different treatment courses can change the responses observed. Whether the combination of the platinum with other drugs makes a difference still needs to be evaluated in a larger sample set, and our classification model helps in evaluation.

In summary, we found an association between reproducible immunological subtypes and prognosis in platinum-assisted chemotherapy for OC. A subset of immunoactivated patients may well benefit from platinum therapy, while for those with subtypes associated with poor prognosis, combination anti-TCGF-beta therapy is recommended instead due to high TGF-beta signaling (22). Our results will provide evidence for clinical decision-making and can help optimize treatment to improve survival outcomes.

## Data Availability Statement

The datasets presented in this study can be found in online repositories. The names of the repository/repositories and accession number(s) can be found in the article/**Supplementary Material**.

## Author Contributions

XM had contributions to the conception and design of the study; YL performed the data analysis, interpretation, and manuscript drafting; HW and MC contributed to the data acquisition and assessment. All authors contributed to manuscript reviewing, approved the final version of the manuscript, and agreed to be accountable for all aspects of the study.

## Conflict of Interest

The authors declare that the research was conducted in the absence of any commercial or financial relationships that could be construed as a potential conflict of interest.

## Publisher’s Note

All claims expressed in this article are solely those of the authors and do not necessarily represent those of their affiliated organizations, or those of the publisher, the editors and the reviewers. Any product that may be evaluated in this article, or claim that may be made by its manufacturer, is not guaranteed or endorsed by the publisher.
