# Supplementary material for: The Immune Subtype Contributes to Distinct Overall Survival for Ovarian Cancer Patients With Platinum-Based Adjuvant Therapy
Source: Front Immunol. 2022 Jun 24;13:872991. doi: 10.3389/fimmu.2022.872991 (PMC9263722; doi:10.3389/fimmu.2022.872991)
Supplement: Supplementary file 1 [file DataSheet_1.docx]

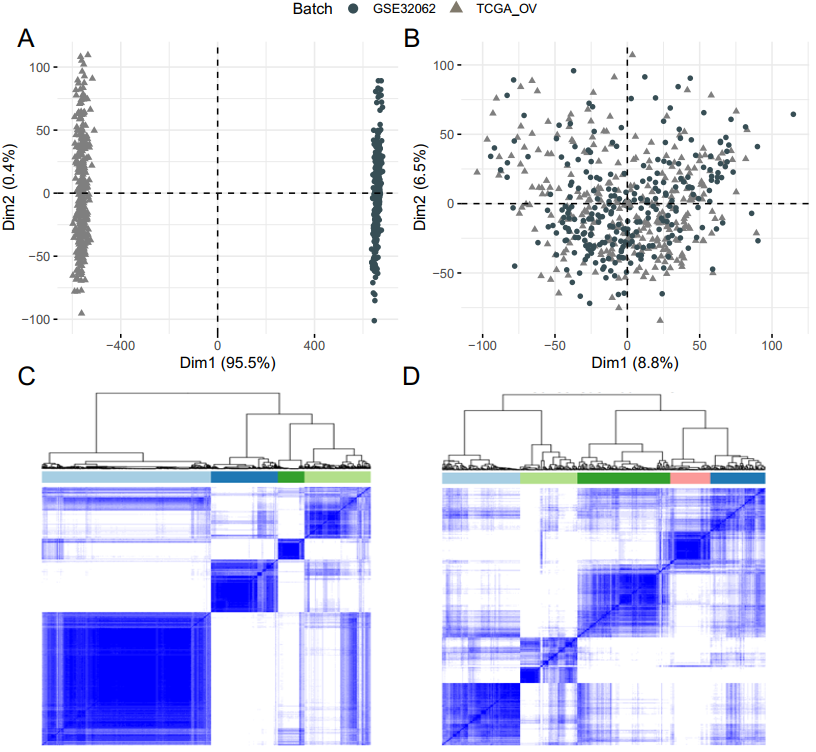


Fig S1. Revisiting immune subtypes in ovarian cancer. Integrated data was used to identify immune subtypes (A) before and (B) after the correction batch. Consensus matrix for (C) immune-related genes and (D) patients derived from consensus clustering analysis.


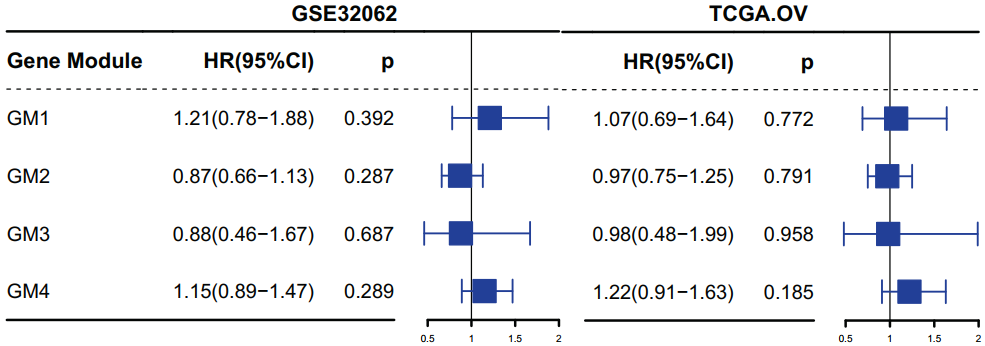


Fig S2. Forest plot of the prognostic effect of gene module scores on predicting PFS in GSE32062 (left) and TCGA (right) cohort.


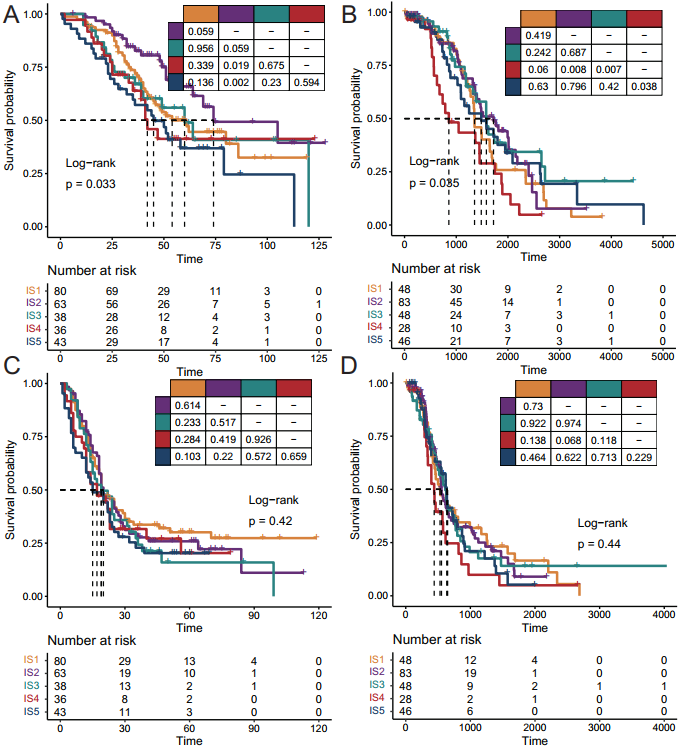


Fig S3. Kaplan–Meier curves show differences in OS for immune subtype patients in the (A) GSE32062 and (B) TCGA cohort, and PFS for immune subtype patients in the (C) GSE32062 and (D) TCGA cohort.


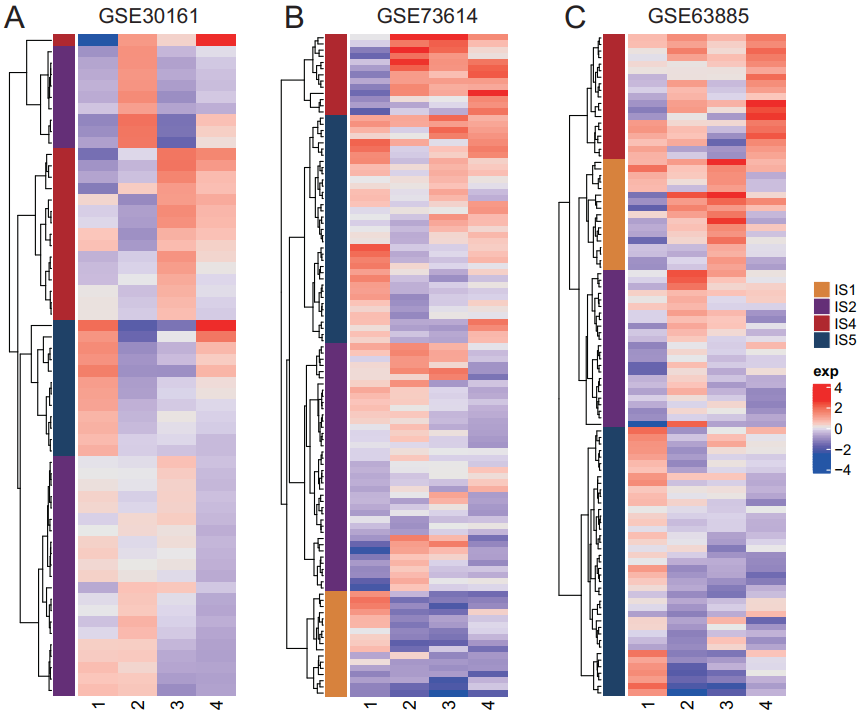


Fig S4. Samples were classified based on the activity of gene modules in three independent validation sets.


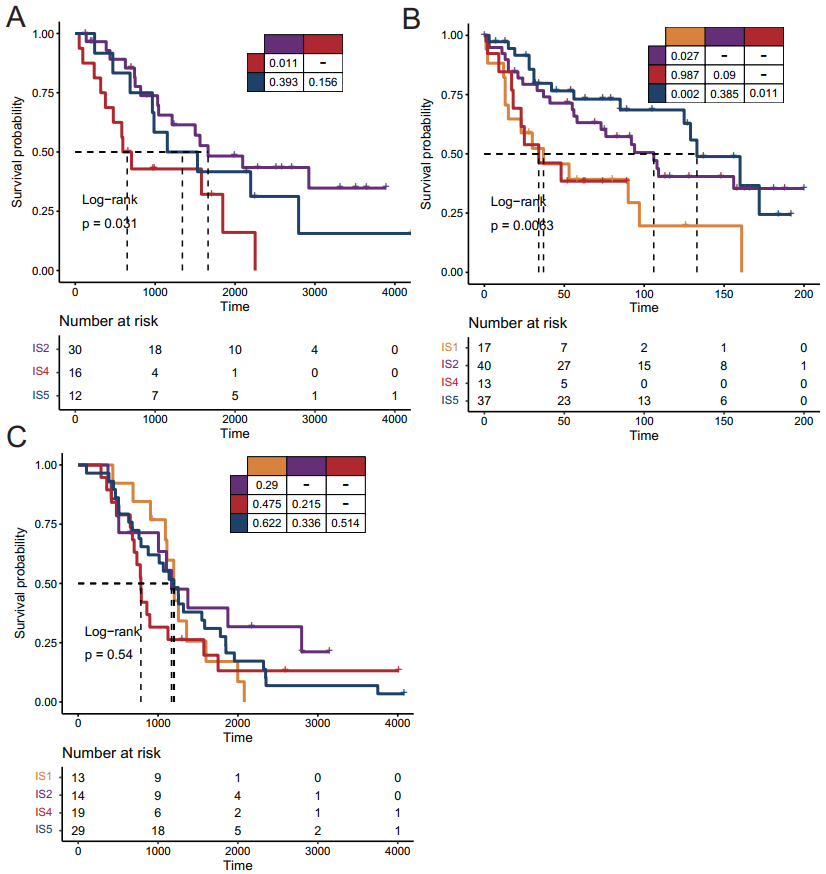


Fig S5. Kaplan–Meier curves for OS of patients in three independent cohorts stratified by the immune subtypes. (A) GSE30161, (B) GSE73614, (C) GSE63885.


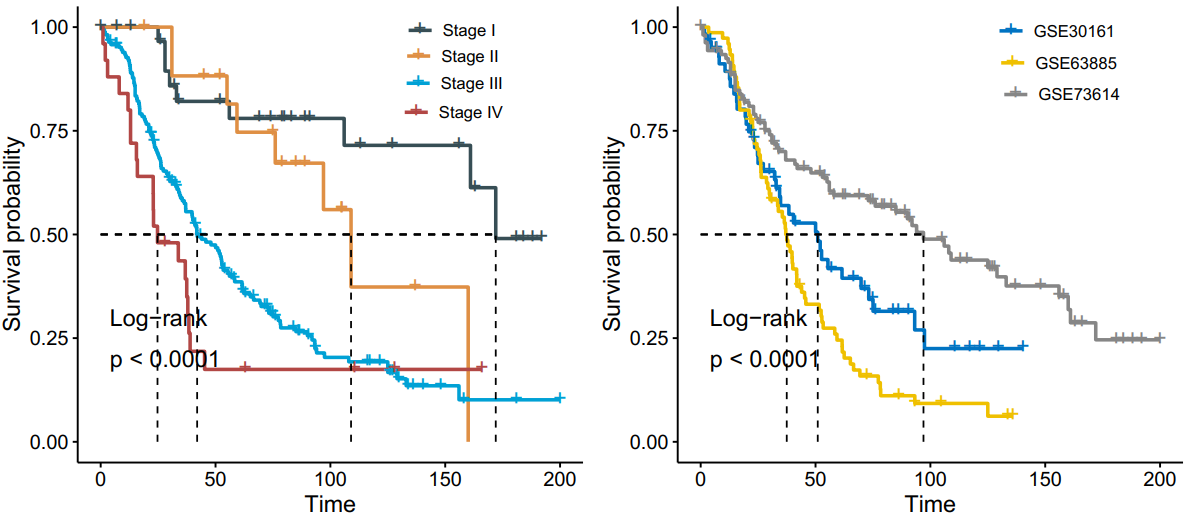
 Fig S6. Kaplan–Meier curves for OS of patients in combined independent cohorts stratified by stage and datasets.


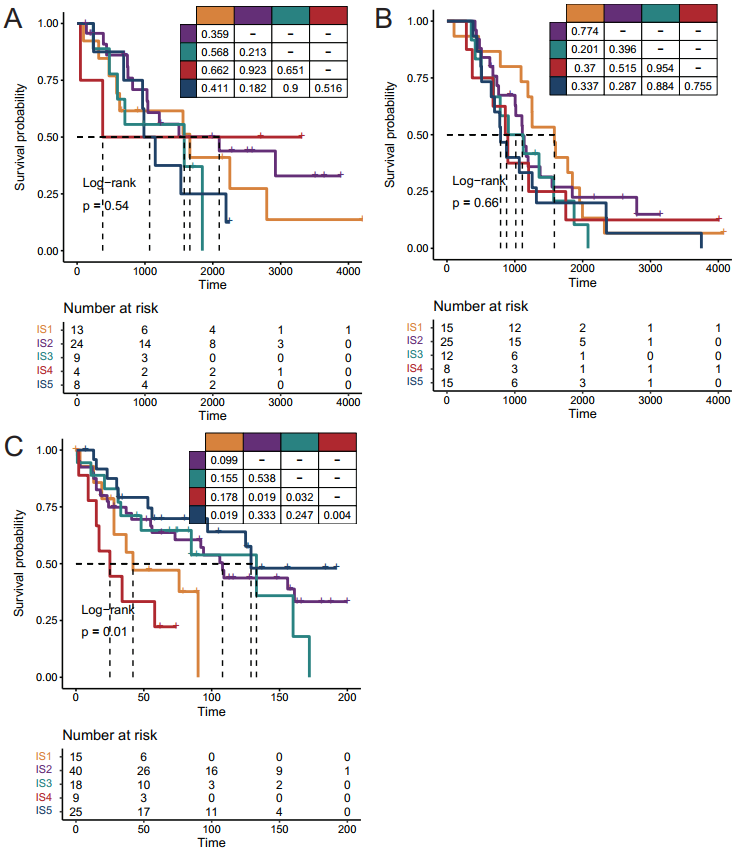


Fig S7. Kaplan–Meier curves for OS of patients in three independent cohorts stratified by the multi-classification logistic model. (A) GSE30161, (B) GSE63885, (C) GSE73614.


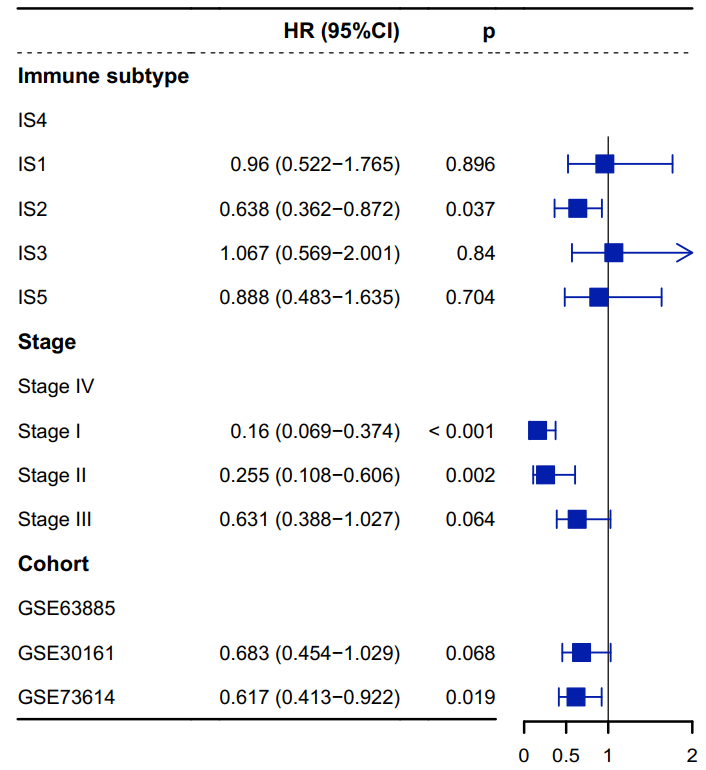


Fig S8. Multivariable Cox regression analysis of OS including immune subtype (stratified by multi-classification logistic model), stage, and datasets in a combined validation cohort. IS4 was used as the baseline for survival risk comparison for the immune subtype variable. Stage IV was used as the baseline for survival risk comparison for the stage variable. GSE63885 was used as the baseline for survival risk comparison for cohort variables.


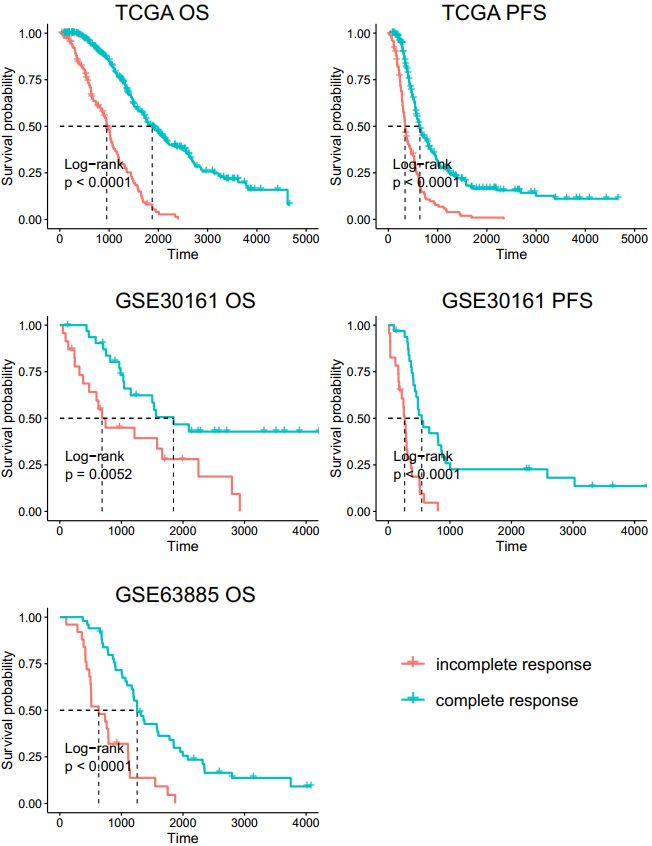


Fig S9. Kaplan–Meier curves for OS and PFS of patients stratified by the clinical response in three cohorts.


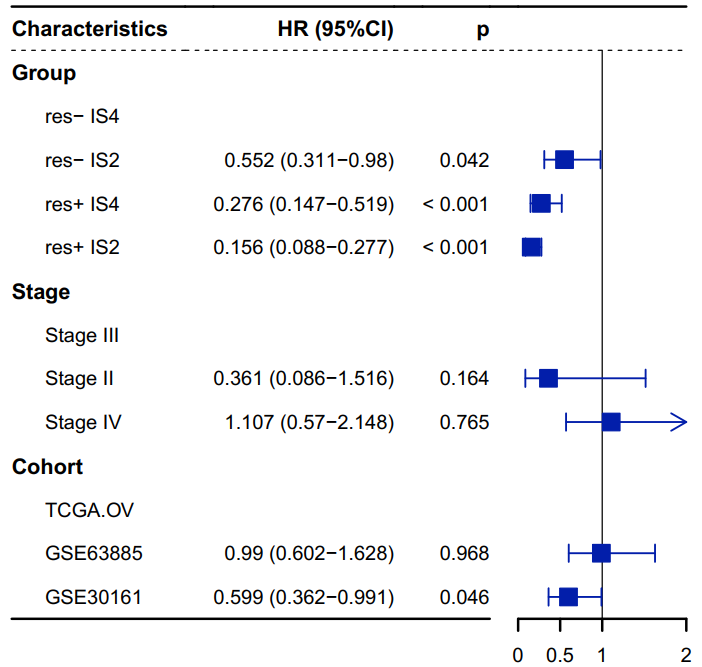


Fig S10. Multivariable Cox regression analysis of OS including a group that combined treatment response and immune subtypes, stage, and cohorts. IS4 patients with incomplete responses were used as the baseline for survival risk comparison for group variables. Stage III was used as the baseline for survival risk comparison for the stage variable. TCGA was used as the baseline for survival risk comparison for cohort variables.


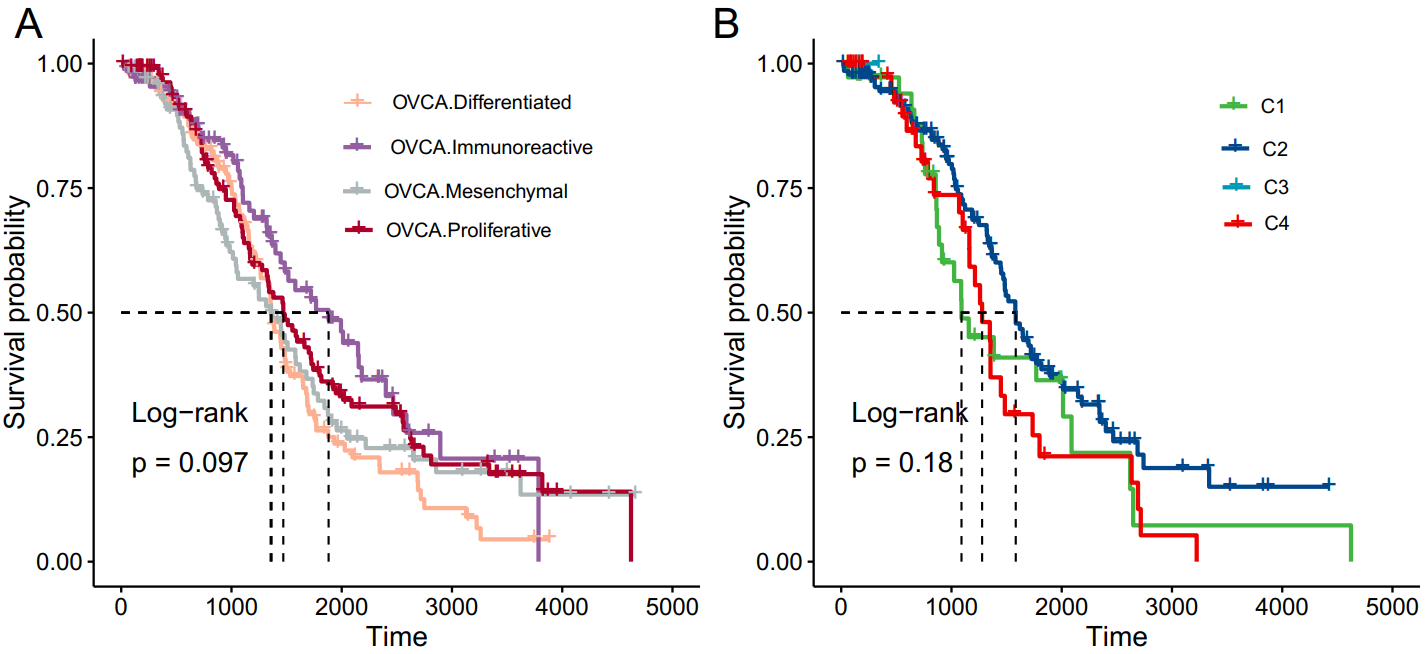
 Fig S11. Kaplan–Meier curves for OS and PFS of patients stratified by the classic ovarian cancer subtypes.


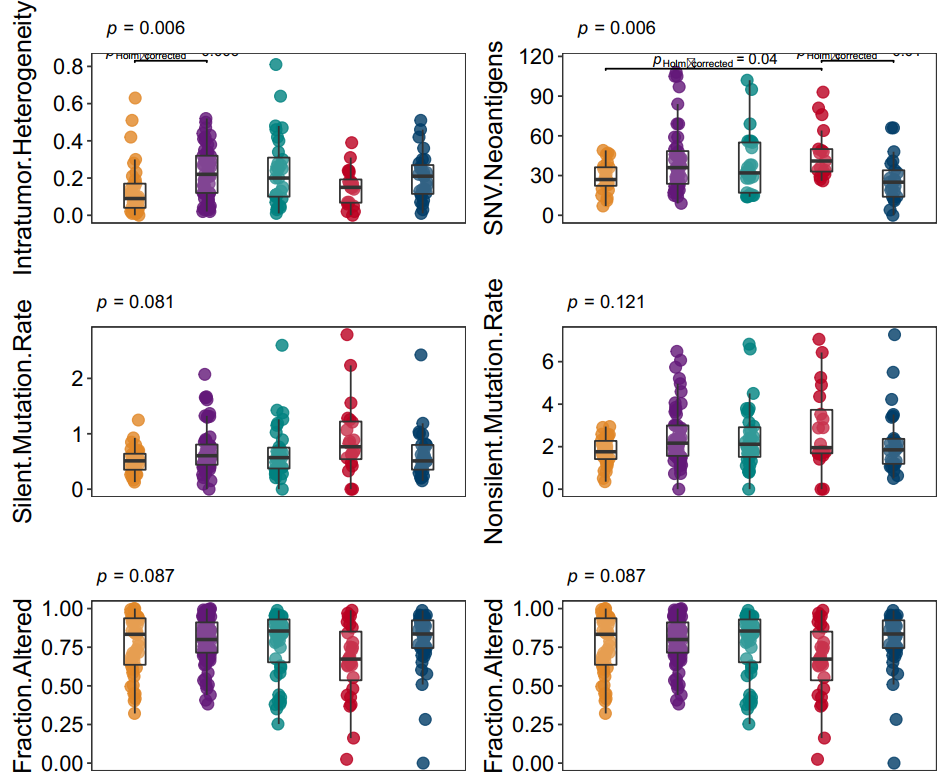


Fig S12. Genomic characters across different immune subtypes.


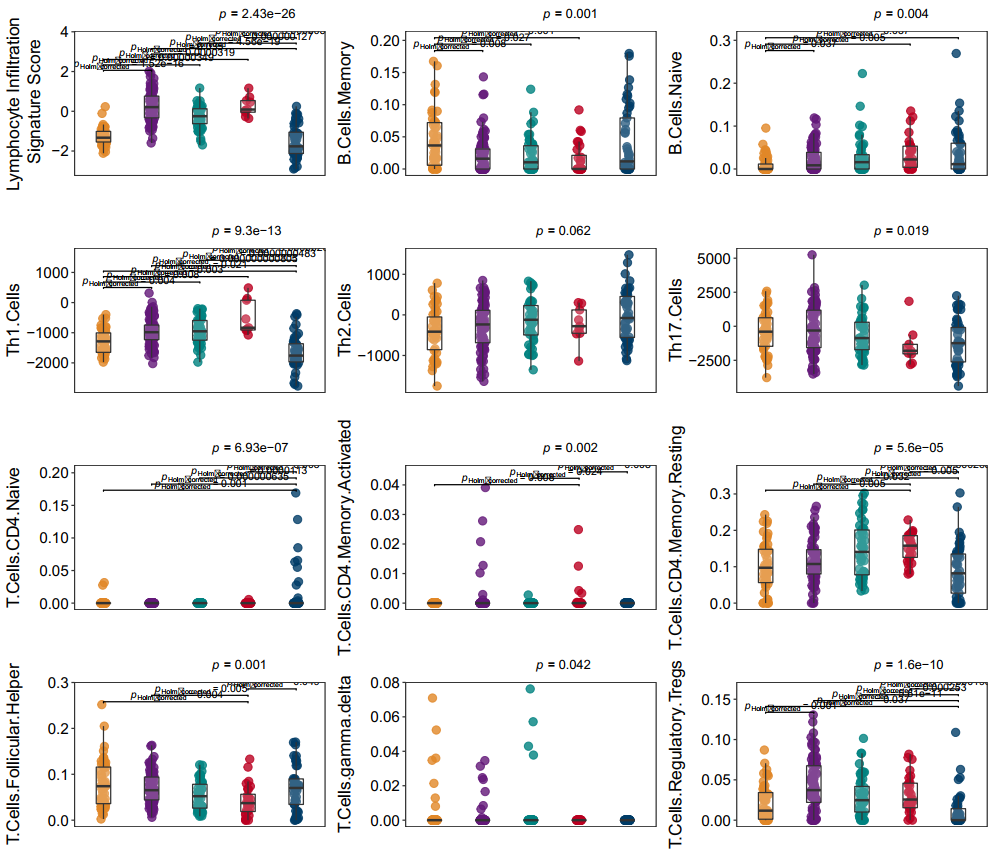


Fig S13. Immune composition across different immune subtypes (part A).


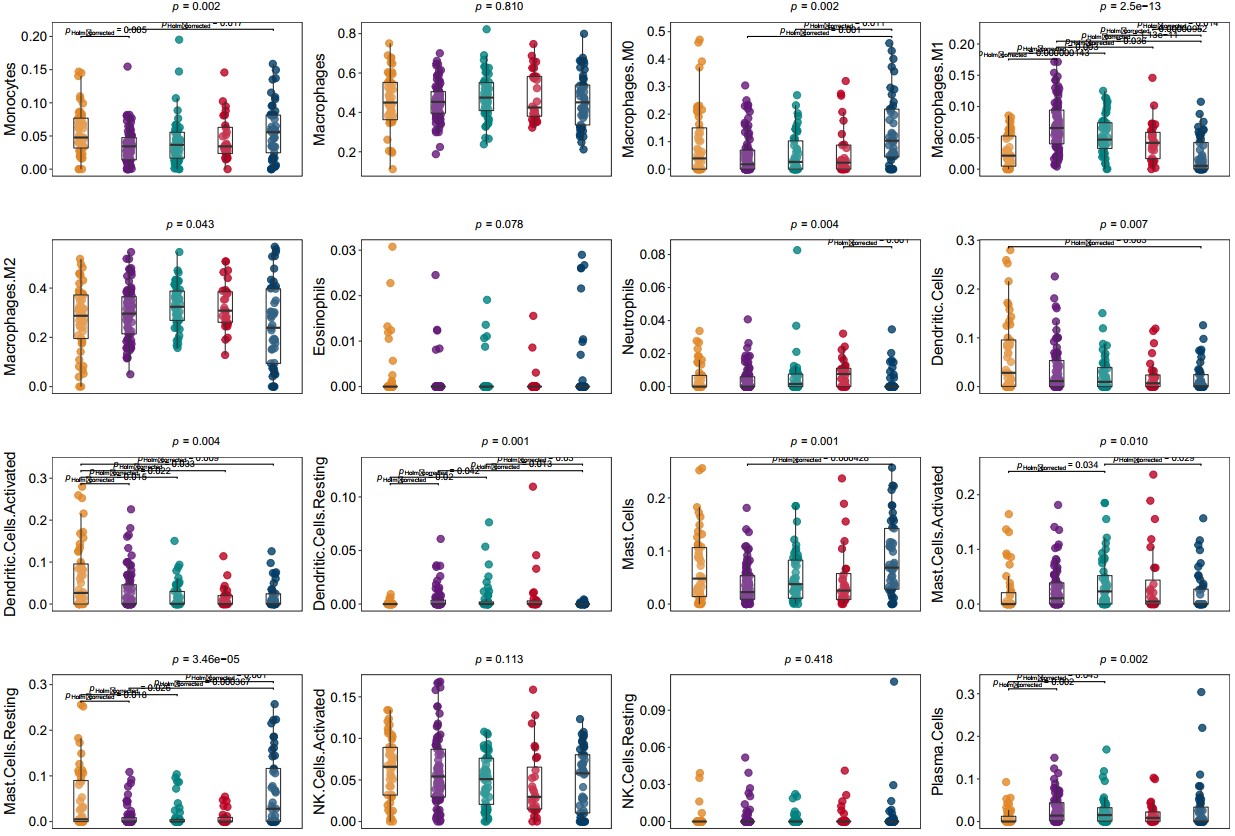


Fig S13. Immune composition across different immune subtypes (part B).


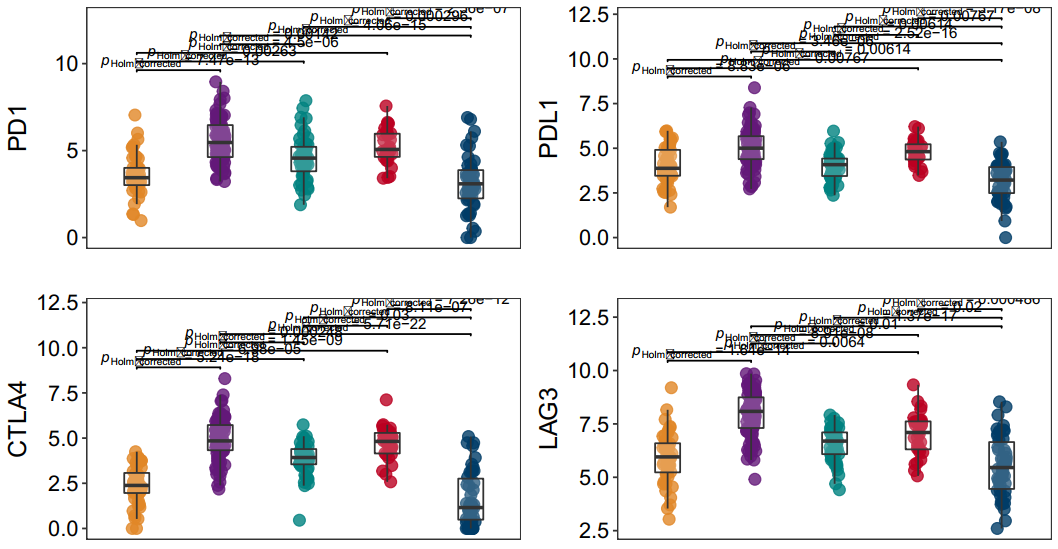


Fig S14. Differential expression of immune checkpoints across immune subtypes.
